# Retinal Vein Occlusion–Background Knowledge and Foreground Knowledge Prospects—A Review

**DOI:** 10.3390/jcm13133950

**Published:** 2024-07-05

**Authors:** Maja Lendzioszek, Anna Bryl, Ewa Poppe, Katarzyna Zorena, Malgorzata Mrugacz

**Affiliations:** 1 Department of Ophthalmology, Voivodship Hospital, 18-400 Lomza, Poland; lendzioszek.majka@gmail.com (M.L.); epoppe@interia.pl (E.P.); 2 Department of Ophthalmology and Eye Rehabilitation, Medical University of Bialystok, 15-089 Bialystok, Poland; malgorzata.mrugacz@umb.edu.pl; 3 Department of Immunobiology and Environment Microbiology, Medical University of Gdansk, Dębinki 7, 80-211 Gdansk, Poland; katarzyna.zorena@gumed.edu.pl

**Keywords:** retinal vein occlusion, macular edema, prevention, diagnosis, treatment, artificial intelligence (AI)

## Abstract

Thrombosis of retinal veins is one of the most common retinal vascular diseases that may lead to vascular blindness. The latest epidemiological data leave no illusions that the burden on the healthcare system, as impacted by patients with this diagnosis, will increase worldwide. This obliges scientists to search for new therapeutic and diagnostic options. In the 21st century, there has been tremendous progress in retinal imaging techniques, which has facilitated a better understanding of the mechanisms related to the development of retinal vein occlusion (RVO) and its complications, and consequently has enabled the introduction of new treatment methods. Moreover, artificial intelligence (AI) is likely to assist in selecting the best treatment option for patients in the near future. The aim of this comprehensive review is to re-evaluate the old but still relevant data on the RVO and confront them with new studies. The paper will provide a detailed overview of diagnosis, current treatment, prevention, and future therapeutic possibilities regarding RVO, as well as clarifying the mechanism of macular edema in this disease entity.

## 1. Introduction

Retinal vein occlusion (RVO) is the second most common retinal vascular disease, surpassed only by diabetic retinopathy. RVO is the vascular occlusion of either the branch or central retinal vein, resulting in potential vision changes and long term sequelae. The global burden of this disease entity is increasing, as evidenced by the new epidemiological data. In 2008, the disease affected 16.4 million people worldwide, while in 2015, it affected 28.06 million people, including 23.38 million individuals suffering from branch retinal vein occlusion (BRVO) and 4.67 million individuals with central retinal vein occlusion (CRVO) [1].

Depending on the anatomical location of the thrombus, the following distinctions are made: CRVO, upper or lower branch retinal vein occlusion (hemi-CRVO; HRVO) and BRVO. In the case of CRVO, occlusion occurs at the level of the lamina cribrosa of the optic nerve, while in the case of HRVO, the presumed site of occlusion is one of the two stems of the improperly divided central retinal vein. BRVO mainly occurs at arteriovenous crossings [2]. CRVO and HRVO can be divided into ischemic and non-ischemic types, that display different pathogeneses, clinical features and prognoses, and have different treatments. Meanwhile, the BRVO consists of major BRVO and macular BRVO, with the most common pathogenesis of occlusion being the presence of a common adventitial sheath at arteriovenous crossings. In situations involving risk factors for cardiovascular diseases, arterial thickening and hardening lead to the mechanical narrowing of the venous vessel wall. Consequently, turbulent blood flow in the venous vessel initiates a cascade of events associated with Virchow’s triad [2]. In the post-occlusive event, inflammation plays an important role in the pathophysiology, progression, and prognosis of RVO. Numerous studies have shown that cytokines and chemokines in the aqueous humour and vitreous body are correlated with the RVO, particularly the interleukin family, VEGF, MMP, LPA-ATX, and PDGF [3]. Another indication of the inflammatory pathophysiology of RVO is an observation that higher neutrophil-to-lymphocyte ratio (NLR) and platelet-to-lymphocyte ratio (PLR) were associated with the development of RVO [4,5].

Vision is the most precious of all the senses, as reflected in the work of Enoch et al., in which 88% of respondents confirmed this opinion. The results of their study suggested that people would choose an average of 4.6 years of perfect health compared to 10 years of life with a total loss of vision [6]. In the case of RVO occurrence, the quality of life related to vision drastically decreases, especially in the case of CRVO [7]. The impact on vision quality, as well as the chronicity of this disease entity, affects mental health due to concerns about the future as well as limitations on the previous roles in social and professional life [7].

Typical risk factors accounting for the development of RVO include age, hypertension, hyperlipidemia, diabetes, atherosclerosis, high body mass index, and smoking (Table 1) [2,8]. Age is a critical risk factor, as over 50% of cases occur in individuals over 65 years of age [9]. Due to shared risk factors accounting for cardiovascular disease, it has been repeatedly shown that patients with RVO have an increased risk of cardiovascular events and all-cause mortality [10]. It has even been possible to determine a timeframe after the onset of RVO when greater attention should be paid to the patient due to the increased risk of ischemic or hemorrhagic stroke, which is the first 30 days following the development of RVO [11]. Retinal microangiopathy is associated with a lacunar stroke, and the frequency of changes in small brain vessels in patients with RVO is 54% [12]. Interestingly, Cho et al. demonstrated that in younger individuals (<60 years old) with the RVO, cerebral small vessel disease (SVD) occurred in 38% of patients compared to 4% in the control group, suggesting that in young individuals, RVO may be a marker of cerebral SVD. Less specific risk factors for RVO include chronic kidney disease, multiple myeloma, polycythemia vera, leukemia, systemic lupus erythematosus, sarcoidosis, syphilis, and thrombotic disorders, such as hyperhomocysteinemia, antiphospholipid syndrome, factor V Leiden mutation, protein C and S deficiency, antithrombin deficiency, or prothrombin gene mutation (G20210A) (Table 1) [2].

Chen, in his work, aimed to identify risk factors for CRVO in younger populations and demonstrated that the most significant risk factors for individuals under 40 years of age were primary open-angle glaucoma, retinal vasculitis, pseudotumor cerebri, hypercoagulability states, and hyperlipidemia [13]. In patients with RVO under the age of 50 without significant cardiovascular risk factors, consideration should be given to testing for thrombophilia or other conditions associated with hypercoagulability [14].

Ophthalmic risk factors for RVO include open-angle and closed-angle glaucoma, hyperopia, central serous chorioretinopathy, retinal vasculitis and short axial length (Table 1) [13,15,16,17,18].

**Table 1:** Systemic and ophthalmological risk factors for RVO [2,8,15,16,17].

Risk Factors for RVO	
Systemic	Ophthalmological
Atherosclerosis: old age, hypertension, hyperlipidemia, diabetes, high body mass index, smoking	open-angle glaucoma and closed-angle glaucoma
Vasculitis: systemic lupus erythematosus, sarcoid, syphilis	hyperopia
Neoplasia: polycythemia rubra vera, multiple myeloma, leukemia	short axial length
Thrombophilic disorders: hyperhomocysteinemia, antiphospholipid syndrome, factor V Leiden mutation, protein C and S deficiency, antithrombin deficiency, or prothrombin gene mutation (G20210A)	central serous chorioretinopathy
	retinal vasculitis

The prognosis in the cases of vision loss caused by BRVO depends on the degree of perfusion deficit and the location of the thrombus. The Branch Vein Occlusion Study (BVOS) research group presented a collection of results regarding the natural course of untreated BRVO [19]. Their meta-analysis indicates that the initial visual acuity (VA) ranges from 20/40 to less than 20/200, with spontaneous improvement over time; however, a VA above 20/40 is rare. An improvement in visual acuity by 2 or more lines was noted in at least 33% of patients, and some studies reported improvement in up to 75% of patients. A systematic review showed that complications in the form of macular edema (ME) affected 5% to 15% of patients but spontaneously subsided in 41% of patients affected by this complication. Neovascular complications such as anterior or posterior segment neovascularisation and neovascular glaucoma are very rare in the case of BRVO. Complications such as vitreous hemorrhage may affect up to 41% of eyes. Importantly, the occurrence of the BRVO in one eye increases the risk of developing this disease entity in the other eye by 10%.

CRVO is classified into non-ischemic (NI-CRVO) and ischemic (I-CRVO) forms, distinguished by fluorescein angiography (FA). The non-ischemic form occurs in about 75% of all CRVO patients and is characterised by the presence of retinal non-perfusion areas smaller than 10 disc areas. Ischemic CRVO is defined by the appearance of neovascularisation on the surface of the iris (rubeosis iridis) or retinal non-perfusion areas confirmed by FA larger than 10 disc areas.

The Central Vein Occlusion Study (CVOS) research group analysed the key data on the natural history of untreated CRVO [20]. Their study showed that the initial VA was poor at the beginning of the study (<20/40) and tended to deteriorate, especially in the case of ischemic CRVO, where VA usually was less than 20/200. Up to 34% of eyes with non-ischemic CRVO progressed to ischemic CRVO within 3 years. The development of non-perfusion and ischemia was fastest within the first four months and progressed throughout the study period. The low initial visual acuity and the degree of non-perfusion observed in FA were the strongest predictors of neovascularisation (NV). In the cases of non-ischemic CRVO, the risk of neovascularisation was up to 33%, and the risk of neovascular glaucoma (NVG) was 0%, while in ischemic CRVO, it ranged from 20% for NV to 23% to 60% for NVG. Patients with CRVO have a high risk of developing ME, which spontaneously subsides in 30% of NI-CRVO patients. Other complications, such as vitreous hemorrhage, occur in 10% of patients. The risk of developing CRVO in the fellow eye is 1.4% within 3 years, and the risk of any RVO in the fellow eye is estimated at 5% within one year.

Further on in this review, we have focused on a detailed analysis of the pathophysiology of ME secondary to retinal vein occlusion, the RVO diagnosis, prevention, and treatment, as well as new diagnostic and therapeutic possibilities.

## 2. Macular Edema in RVO

Macular edema is a serious complication of many diseases widely described in the related literature, such as diabetes, retinal vein occlusion, choroidal neovascularisation, choroiditis, central serous chorioretinopathy, or rarer ones like Irvine–Gass syndrome, idiopathic macular telangiectasia type 1 and 2, paclitaxel-induced maculopathy, MEK inhibitor-associated maculopathy, or maculopathy associated with hypoproteinemia [21,22,23,24,25,26]. Macular edema within the course of RVO affects 3 million people worldwide and is the leading cause of vision loss in the patients with retinal vein occlusion [27]. Symptoms include a central vision loss, blurred vision, metamorphopsia, micropsia, and decreased contrast or colour sensitivity. 

Macular edema occurs extremely often in the cases of CRVO [20]. Furthermore, in their meta-analysis, McIntosh et al., demonstrated that only 30% of macular edema cases in non-ischemic CRVO spontaneously subsided. In the case of BRVO, macular edema occurs in 15% to even 30% of cases [19,28]. Within the framework of the meta-analysis conducted by Rogers et al., it was determined that macular edema associated with BRVO subsided in 18% of eyes after 4.5 months from the onset of occlusion symptoms, reaching 41% after 7.5 months [19].

Fluid accumulation results from the dysregulation of the processes responsible for fluid entry and exit, and is driven by the Starling equation when the internal or external blood–retinal barrier is disrupted [29]. This allows for the infiltration of retinal tissue by proteins and other dissolved substances, resulting in an increase in tissue osmotic pressure, which leads to the accumulation of extracellular fluid. The internal blood–retinal barrier consists of endothelial cells and their intercellular junctions, pericytes, and retinal glial cells. The latter include microglia and Müller glial cells (RMG) and astrocytes. The external blood–retinal barrier is formed by the retinal pigment epithelium and the complex of its intercellular junctions and outer limiting membrane (OLM) [30]. Damage to any of those barriers, that cannot be counteracted by defensive mechanisms, leads to an increased fluid permeability across the blood–retinal barrier, reduced drainage functions for which the glia and retinal pigment epithelium are responsible, and protein leakage [31]. 

Fluid can accumulate diffusely in the central retina, within cysts usually located in the inner nuclear layer and Henle’s fibre layer or in the subretinal space [32]. In the case of RVO, cystoid macular edema and subretinal fluid accumulation are most common [33]. The visual consequences of macular edema mainly depend on structural changes caused by the accumulation of intraretinal or subretinal fluid, with a poorer prognosis associated with changes in external retinal structures, such as the OLM or photoreceptor segments [34,35].

Given the current considerations regarding macular edema, the particular attention is focused on glial cells, the processes of which wrap around the retinal capillaries, especially on RMG. Müller glial cells are the only cells spanning the entire thickness of the neurosensory retina, providing the contact between all types of retinal nerve cells, retinal vessels, and the vitreous cavity. Importantly, the processes of RMG are ongoing at all the levels of retinal vascular plexuses: superficial, intermediate, and deep, whereas astrocytes are present only at the level of the superficial plexus [36]. The unique structure of Müller cells facilitates the structural, metabolic, and homeostatic support to the retinal neurons [37]. Müller glia also influence synaptic activity in the retina through the recycling of neurotransmitters, including glutamate and gamma-aminobutyric acid (GABA) [38]. Müller cells are among the key participants in controlling water and ion homeostasis in the interstitial areas of the retina. This principal retinal glial cell buffers changes in the intracellular potassium concentration mainly through Kir2.1 and Kir4.1 channels, which consequently leads to the flow of potassium ions through aquaporin 4 channels (AQP4) [37,39,40]. The retina is characterised by a high degree of metabolism, resulting in the production of water and carbon dioxide, which are eliminated by Müller cells. Fulfilling this role requires the Müller cells to possess active carbonic anhydrase that participates in water transport, carbon dioxide economy, and bicarbonate transport, thereby directly influencing pH regulation [41,42]. In addition, Müller glial cells have the ability to produce pro-inflammatory and anti-angiogenic substances. Behzadian et al. demonstrated that the production of transforming growth factor β (TGF-β) by glial cells stimulated the production of matrix metalloproteinase (MMP-9), leading to the increased permeability of endothelial cells and contributing to the breakdown of the blood–retinal barrier [43]. On the contrary, Hauck et al. demonstrated the protective effect of Müller glial cells on the retinal vascular endothelium by producing pigment epithelium-derived factor (PEDF), which inhibited the permeability induced by VEGF through several mechanisms [44]. The most important mechanisms include inhibiting the phosphorylation and activation of five major signalling intermediates of VEGF-A, namely phosphoinositide-3-OH kinase (PI3K), AKT, focal adhesion kinase (FAK), Src (Y416), and phospholipase C gamma (PLC-γ), as well as activating γ-secretase to remove the ectodomain of VEGF-R2. Additionally, it involves inhibiting the transcriptional activation of beta-catenin, which links the intracellular cadherin domain with actin filaments, the main component of the cytoskeleton [45,46,47].

The mechanism of diabetic macular edema formation has been very well elucidated [30]. The cellular mechanisms underlying the macular edema associated with RVO are relatively poorly understood. Dominguez et al. demonstrated that acute macular edema in an experimental BRVO model in mice was associated with a wave of endothelial cell (EC) apoptosis, followed by their proliferation and resolution of the edema, and in the final phase, a sustained loss of pericytes (PC) and a continued increase in EC apoptosis and proliferation, involving TGFβ, TSP-1 as well as FGF2 [48]. By the end of the 20th century, the loss of pericytes (PC) was demonstrated in an experimental BRVO model in monkeys, suggesting that similar phenomena may occur in human eyes [49]. In the study by Celik et al., 34% of the patients with macular edema secondary to BRVO showed retinal swelling in the form of serous retinal detachment (SRD), and furthermore, all the patients with SRD also presented with cystoid macular edema (CME). This could be indicative of damage to the retinal pigment epithelium (RPE) barrier; however, they did not observe any defects in the outer retinal surface [50]. The decrease in potassium conductivity, caused by the reduction in Kir4.1 and aquaporin levels, as well as the improper localisation of Kir4.1 protein and osmotic swelling of Müller cells, resulting in the formation of retinal edema, was observed in a BRVO rat model [51]. Biological factors, including cytokines and angiogenic factors, detected in the patients with macular edema in the course of the RVO include: interleukin-1β (IL-1β), IL-6, IL-8, IL-12, IL-15, IL-17, IL-23, TGF-β, VEGF, basic fibroblast growth factor (bFGF), chemokine CXC motif ligand 10 (CXCL-10), chemokine ligand 2 (CCL2), platelet-derived growth factor (PDGF-AA), serum amyloid A (SAA), soluble intercellular adhesion molecule-1 (sICAM-1), soluble vascular endothelial growth factor receptor 2 (sVEGFR-2) (Table 2) [30]. The large number of biological factors involved in the development of macular edema in the case of RVO provides for the opportunity to target therapy at more mediators than just VEGF.

There are several hypotheses explaining why the macula is particularly susceptible to edema in retinal vascular diseases as compared to other retinal structures. The foveal centre consists exclusively of densely packed cone photoreceptors, an avascular zone, and specific foveal Müller cells, the density of which at this location is five times higher than at the periphery [52]. In the foveal pit region, Müller cells are characteristically elongated in a Z-shaped pattern, which facilitates their binding to photoreceptor axons through connecting proteins such as occludin or zonula occludens-1 (ZO-1), preventing protein accumulation [53]. Omri et al. suggested that the dysfunction of those specific Müller cells would lead to the disruption of those connections, resulting in protein accumulation and consequently water influx. In his meta-analysis, Daruich proposed the existence of a glymphatic system in the fovea, explaining it by demonstrating strong AQP4 expression along the Müller cells in a Z-shaped pattern [30]. That thesis could be supported by the findings of a study conducted by Mathieu et al., who presented histological evidence for the existence of a glymphatic pathway in the optic nerve utilising AQP4 [54].

## 3. Diagnostics

The advancements in technology allow for the utilisation of various imaging studies, which enhances the speed of diagnosis and optimises patient care. In the case of RVO, diagnosis is typically based on fundus examination aided by the colour fundus photography. Answers to questions such as whether there is macular edema or if there is retinal ischemia, and if so, what its extent is, can easily be provided by optical coherence tomography (OCT), optical coherence tomography angiography (OCTA) as well as fluorescein angiography (FA) and its wide-field variant (UWF-FA), which have been used for many years

Symptoms observed in the fundus of the eye, depending on the location of occlusion, include tortuous and dilated retinal veins, intraretinal hemorrhages, cotton-wool spots, macular edema, or optic disc edema. Late features of RVO may include hard exudates, microaneurysms, venous sclerosis, arterial narrowing, vitreous hemorrhage (VH), tractional retinal detachment (TRD), and neovascularisation of the retina, optic disc, or iris (Table 3) [55]. Among the very rare changes observed in the course of RVO are occlusion of the cilio-retinal artery and whitening of the retina around the veins, considered severe acute macular neuroretinopathy (PAMM) [56,57].

### 3.1. OCT

OCT is the most commonly used imaging method in the case of RVO. One of the main features of the OCT typically used in assessing disease activity and progression is central retinal thickness (CRT). The CRT has been the primary endpoint in most randomised clinical trials: its increase correlates with functional loss, while its decrease correlates with functional improvement. However, in the patients treated with anti-VEGF for macular edema associated with the CRVO or HRVO, a thinner retina does not always correlate with better VA [58]. Other features that can be observed in the course of the RVO by means of the OCT include: subretinal fluid (SRF), intraretinal fluid (IRF), the presence, location, and quantity of hyperreflective foci (HF), the integrity of the ellipsoid zone, disorganisation of Retinal Inner Layers (DRIL) and posterior vitreous detachment (PVD).

The presence of subretinal fluid (SRF) in retinal vein occlusion (RVO) is a topic of debate regarding its predictive value. While the SRF is commonly present at the onset of treatment, its significance as a prognostic factor for visual improvement in the case of RVO remains uncertain. Etheridge et al. evaluated its presence as a negative prognostic factor for visual improvement, contrasting with the findings arising from the study conducted by Lloyd Clark et al., who demonstrated that SRF at the beginning of the study was associated with faster visual improvement. Therefore, the predictive value of the SRF in the case of RVO requires further investigation and clarification [59,60].

The mechanism of HF formation remains elusive; however, their presence in diabetic macular edema (DME) and RVO may suggest a potential association with both inflammation and increased vascular permeability in their pathophysiology [61]. In a study conducted by Chatziralli et al., it was shown that hyperreflective foci were associated with worse visual outcomes, most likely due to damage to the photoreceptor layer. However, their reduction can be achieved through treatment with anti-VEGF agents or steroids [61]. Kang et al., analysed the impact of the depth of HF on best-corrected visual acuity (BCVA) during anti-VEGF treatment and found that the presence of HF in the outer retinal layers was associated with poorer therapeutic outcomes [62].

DRIL refers to a condition in which the differentiation between inner retinal layers becomes impossible. In the OCT imaging for DRIL, we cannot detect boundaries between the ganglion cell–inner plexiform layer, inner nuclear layer, and outer plexiform layer. This important biomarker reflects the degree of damage to cells accompanying macular edema, and its morphological extent has been correlated with the degree of vision loss, especially in diabetic retinopathy [63]. Mimouni et al. concluded that changes in the extent of DRIL after the initial 3-month injections indicated eyes with a high likelihood of subsequent improvement or a worsening of BCVA [64]. Babiuch et al., similarly, observed that the initial presence of DRIL and changes in its morphology during therapy for macular edema secondary to RVO could be useful biomarkers for improving VA outcomes, especially in the cases of CRVO/HRVO [65]. An early recovery within 3 months, both in terms of DRIL and EZ (ellipsoid zone) parameters, is a key factor influencing the one-year VA outcomes [66]. On the other end of the spectrum, there are the studies that have not shown any OCT parameters, including DRIL, which serve as future predictors of VA in RVO. Only baseline VA and age have been correlated with the subsequent BCVA [59,67].

The OCT allows for a precise assessment of the connections between the vitreous body and the retina. In eyes with complete PVD, the incidence of ME in CRVO and neovascularisation is often lower than in the patients with incomplete PVD [68].

### 3.2. OCTA

The angio-OCT technique allows for the evaluation not only of the superficial vascular plexus but also of the deep retinal capillary plexus, as well as the assessment of the foveal avascular zone (FAZ) and choroid. Samara et al. demonstrated that reduced vessel density in both the superficial and deep retinal vascular networks in BRVO and the enlargement of the FAZ correlated with visual acuity loss [69]. The enlargement of the FAZ in eyes with RVO reflects macular ischemia and may serve as a reliable biomarker of visual disturbances [70,71,72]. Moreover, in the cases of retinal vein occlusion, cystoid spaces co-localise in areas where there is a loss of deep capillary plexuses. After the resolution of secondary ME due to RVO, there is no regeneration of this area, which increases the tendency for edema recurrence in the same location [31]. Suzuki et al. revealed the presence of collateral vessels in both the superficial and deep vascular plexuses during the acute phase of BRVO. Furthermore, they found that the presence of collaterals accelerated the reabsorption of edematous fluid, while not affecting BCVA. The possibility of developing microaneurysms within these vessels may be a risk factor for treatment-resistant macular edema in eyes with BRVO [73].

### 3.3. OCT-Leakage

Cunha-Vaz et al. introduced a novel method based on the OCT called OCT-Leakage, which accurately determines the sites of blood–retinal barrier damage by mapping areas with lower than normal optical reflectivity, thereby reflecting changes in extracellular fluid within the retina [29]. This study facilitates the identification of areas with increased extracellular fluid (intraretinal and subretinal) in different layers of the retina, thus directly identifying retinal cells that are more affected than others, allowing for the introduction of targeted treatment methods. Farinha et al. confirmed the complementarity of that method with the OCTA, which is highly promising for a better understanding of the pathophysiology of macular edema in the case of RVO and, consequently, for optimal patient management with this condition [74].

### 3.4. Fluorescein Angiography (FA)

The typical findings observed in fluorescein angiography in a patient with RVO include a delayed filling of the occluded retinal vein and a lack of perfusion in the capillary vessels. Intraretinal hemorrhages may cause dye blockage, and late leakage (hyperfluorescence) may be the result of ME or retinal neovascularisation.

That diagnostic method is helpful in differentiating ischemic RVO from non-ischemic RVO. The lack of perfusion in the peripheral retina characterises ischemic RVO and is an important risk factor for the development of neovascularisation. Angiography allows for the identification of such areas, aiding in effective laser treatment. The lack of contrast flow through the macular capillaries is associated with the poor visual prognosis and lack of visual acuity improvement despite the ME treatment.

New hope comes with ultra-widefield fluorescein angiography (UWF-FA), that allows for the concurrent visualisation of the vascular system in the same angiographic phase up to 200 degrees of the retina, covering 80% of its surface area. Shah et al., utilising the UWF-FA, detected a very high incidence of anatomical changes in the peripheral retinal vessels, even though normal peripheral perfusion had been expected in those patients, indicating better diagnostic capabilities as compared to the FA [75]. According to Thomas et al., the ischemic index (ISI), calculated based on the ratio of non-perfused retina to the visible retina on UWF-FA images, may serve as a prognostic factor for the occurrence of ischemic CRVO in the first year of patient observation. Classifying the CRVO as ischemic based on ISI > 35% is sensitive and specific; however, this finding requires further investigation [76].

The fluorescein angiography is an invasive imaging method due to the need for intravenous injection of a fluorescent dye, which may carry negative consequences [77]. The fluorescein angiography used to be the primary method for imaging retinal vessels before the emergence of the OCTA. However, the demand for that diagnostic method has decreased due to a reduced interest in grid laser treatment associated with the development of anti-VEGF agents, the inability to visualise the deep vascular plexus, and numerous situations where fluorescein vascular leakage impedes the insight into deeper retinal structures [31]. However, that method continues to be a precious diagnostic tool.

## 4. RVO Prevention—Diet

Before we begin to analyse the available treatment options for RVO and its complications, it is worth mentioning preventive measures that may help prevent the onset of that disease.

There are studies demonstrating the impact of diet on the development of retinal vein occlusion [78]. A plant-based diet (PBD) and a Mediterranean diet (MD) are well-known tools for controlling cardiovascular risk factors such as hypertension, hypercholesterolemia, diabetes, and obesity, and as such, they may be desirable nutraceuticals for preventing the occurrence of retinal vein occlusion [79,80]. Moreover, they belong to anti-inflammatory diets that reduce the levels of CRP, TNF-α, TNFR-60, VEGF, MMP-9, MCP-1, PAI-1, sICAM-1, IL-1, IL-6, and many other interleukins, thus exhibiting antithrombotic effects [81]. Single micro and macronutrients may also act protectively against retinal vein occlusion. The related scientific literature provides many examples of the co-occurrence of central retinal vein occlusion with iron deficiency anemia (IDA) [82,83,84]. Iron deficiency is the most common nutritional deficiency, usually resulting from an inadequate diet. The mechanism of thrombosis in IDA involves reactive thrombocytosis and the decreased deformability of red blood cells due to their microcytic structure [85]. Hyperhomocysteinemia, often resulting from deficiencies in its cofactors such as vitamin B12, B6, or folic acid, is another modifiable risk factor for retinal vein occlusion [86,87,88]. On the other hand, a recent meta-analysis found that folate levels were decreased in patients with RVO compared to healthy controls, but not vitamin B12 levels [89]. Studies have shown a strong association between hyperhomocysteinemia and the induction of inflammatory factors, including the expression of adhesion molecules, endothelial dysfunction, oxidative stress, leukocyte adhesion, and the reduced bioavailability of nitric oxide [90,91]. Another widely discussed risk factor for large vessel thrombosis is vitamin D deficiency [92]. There are individual studies describing decreased levels of vitamin D in patients with retinal vascular occlusion [79]. However, an increased susceptibility to RVO in the winter months as well as the functional improvement of the macula in CRVO-related edema after vitamin D supplementation during bevacizumab treatment strongly support such a thesis [93,94]. Vitamin D prevents endothelial damage by exerting anti-inflammatory effects through the inhibition of NF-kappaB activation and antioxidative effects by inhibiting the generation of superoxide anions, inducing NO production, and maintaining mitochondrial function. It also exerts antithrombotic effects by increasing thrombomodulin (TM) expression and downregulating tissue factor (TF) expression [92]. Furthermore, it has been shown that the dietary intake of vitamins A, C, and potassium may have a beneficial effect on the venous profile of the retina by preventing the enlargement of retinal venules [95].

The gut microbiota is currently regarded as an “organ” containing about 150 times more genes than the entire human genome [96]. There is ample evidence confirming the influence of dysbiosis (an imbalance in the gut microbiome composition) on the development of obesity, diabetes, metabolic syndrome, atherosclerosis, inflammatory bowel disease (IBD), gastrointestinal cancers, and even psychiatric disorders [97,98]. Many studies also confirm the association between abnormal gut microbiota and eye diseases [99,100,101,102]. Moreover, Nadeem et al. demonstrated in their study the involvement of gut microbiota in controlling the retinal transcriptome—mainly regulating pathways such as AMPK, VEGF, HIF-1, IGF-1, and MAPK, as well as affecting longevity, oxidative stress, and mitochondrial biogenesis. This suggests the presence of a gut–retina axis [103].

There are studies confirming the influence of the gut microbiome on the development of retinal vascular diseases that have diverse pathophysiology. However, they share common risk factors, such as diabetic retinopathy or retinal artery occlusion [104,105,106]. No direct assessment of the gut flora’s impact on RVO has been undertaken, but there is the suspicion that dysbiosis may increase the likelihood of RVO occurrence by promoting major risk factors for cardiovascular diseases, such as hypertension, atherosclerosis, diabetes, obesity, or dyslipidemia [107]. In the patients with the aforementioned risk factors for the RVO, an increased abundance of Collinsella has been observed (Actinobacteria class), as well as *Escherichia coli*, *Clostridium* species, *Bacteroides caccae*, *Eggerthella lenta*, *Klebsiella*, *Prevotella*, *Desulfovibrio*, or *Parabacteroides* [108,109].

Gut bacteria interact through their metabolites, some of which, such as lipopolysaccharides (LPS) or trimethylamine N-oxide (TMAO), have pro-inflammatory effects, while others, like bile acids (BA) or short-chain fatty acids (SCFA), exhibit protective effects, including neuroprotective, hypotensive, or lipid metabolism-regulating properties. Dysbiosis disrupts the balance between these metabolites, leading to a chronic inflammatory state [107]. Promising results have been obtained in the treatment of type 1 diabetes using fecal microbiota transplantation (FMT) from healthy donors, resulting in a decrease in endogenous insulin production in recipients [110]. Diet is a crucial tool in managing risk factors for RVO and can influence the composition of the gut microbiota. Ni et al. demonstrated that a diet rich in fibre and high-quality protein leads to the increased abundance of bacteria such as Dubosiella, Parasutterella, Bifidobacterium, Muribaculum, Allobaculum, and Ruminococcaceae, reducing hyperglycemia and insulin resistance [111]. It has been demonstrated in a mouse model that intermittent fasting increases the abundance of gut microbiota, which exerts a protective effect on the retina affected by diabetic retinopathy by producing tauroursodeoxycholate (TUDCA), activating the TGR5 receptor [112]. There are numerous reports indicating that the composition of the gut microbiome is disrupted due to the excessive use of antibiotics [113]. Klein et al. estimated that between 2000 and 2015, antibiotic consumption expressed in defined daily doses (DDD) increased by 65% (from 21.1 to 34.8 billion DDD), and the antibiotic consumption rate increased by 39% (from 11.3 to 15.7 DDD per 1000 inhabitants per day) [114]. The significant and global problem of uncontrolled growth in the indiscriminate consumption of antibiotics is highlighted by the fact that during the 71st United Nations General Assembly, the World Health Organization recommended that all member countries collect and report data on antibiotic consumption [115,116]. As part of the preventive measures to protect against the occurrence of retinal vascular diseases, the responsible use of antibiotics and supplementation with probiotics during periods of dysbiosis should be considered. Singh et al. presented evidence of the protective effects of Lactobacillus rhamnosus GG, which was often included in probiotics to mitigate adverse vascular remodelling in the retina under the conditions of hyperhomocysteinemia, which was one of the risk factors for RVO [117]. 

## 5. Treatment

Currently, there are no treatment methods that can safely and effectively reverse occlusion in RVO. Ophthalmic therapies focus on preventing or treating the potential complications of RVO that threaten vision loss. The main benefit of performing basic laboratory tests or measuring blood pressure in the course of RVO is the detection of systemic diseases, with an emphasis on cardiovascular diseases, and the possibility of initiating targeted treatment for the underlying chronic condition. O’Mahoney et al., in their meta-analysis comprising 21 observational studies, noted that the risk of developing RVO increased by 3.5 times with the coexistence of arterial hypertension, by 2.5 times with the coexistence of hyperlipidemia, and only moderately with the coexistence of diabetes. However, the data did not allow for a conclusion regarding whether lowering blood pressure, lipid levels, or glucose levels improves the natural course of RVO [118]. In addition to focusing on the visual organ, ophthalmologists should involve primary care physicians or internists to identify coexisting risk factors and initiate optimal treatment. Important ophthalmic organisations emphasise such multidisciplinary approaches to the patients with RVO [119,120] (Table 4).

### 5.1. Antithrombotic Therapy

One of the proposed methods of RVO treatment is anticoagulant therapy; however, the guidelines regarding this matter contain conflicting recommendations due to inconsistent and limited data from observational studies and individual randomised controlled trials [17,120,121]. Houtsmuller et al. demonstrated that visual acuity improvement was significantly greater with ticlopidine in the patients with newly diagnosed RVO, with its effect being most pronounced in the patients with increased platelet aggregation [122]. Another study revealed that antiplatelet drugs significantly reduced the risk of RVO recurrence as compared to no anticoagulant therapy, by 7.2% with aspirin and 9.1% with ticlopidine, respectively [123]. Another group of drugs that could be considered, considering the pathophysiology of RVO, are low molecular weight heparins (LMWH). Lazo-Langner et al. presented the results of a meta-analysis, suggesting the 78% reduction in the risk of adverse ocular outcomes in RVO with the use of LMWH and the advantage in improving visual acuity as compared to aspirin. They demonstrated that in this particular case, the use of this medication was safe and may not be associated with an increased risk of vitreous hemorrhage [124]. The Antithrombotic Therapy (mainly LMWH) is associated with a greater improvement in visual acuity as compared to the antiplatelet therapy, with 64% vs. 33% of patients, and a lower number of RVO recurrences as compared to the antiplatelet therapy, with 7% vs. 15%, at the expense of an acceptable rate of bleeding complications [125]. In a cohort study involving nearly 300,000 patients with non-valvular atrial fibrillation, the use of non-vitamin K-antagonist oral anticoagulant (NOAC) was associated with a higher risk of retinal vein occlusion as compared to warfarin [126]. Koizumi et al. identified the use of aspirin and warfarin as independent risk factors for CRVO [127].

### 5.2. Photocoagulation Therapy

The treatment with panretinal photocoagulation (PRP) is attributed to the destruction of under-perfused photoreceptors by light energy, causing their mitochondria to cease oxygen intake. It leads to an improved oxygenated blood flow to the inner retina, the reduced production of growth factors—mainly VEGF—and the decreased dilation of capillaries, consequently inhibiting neovascularisation [128]. Before the introduction of intravitreal therapies, focal laser photocoagulation was considered the gold standard for treating macular edema caused by the BRVO. The Branch Vein Occlusion Study demonstrated that the macular grid laser significantly improved visual acuity in the eyes, with BRVO and vision reduced due to macular edema to 20/40 or worse [129]. In the era of available anti-VEGF therapies, the focal laser therapy for ME associated with the BRVO should be considered as a second-line treatment. The Central Vein Occlusion Study did not show any improvement in visual acuity in the case of the macular grid laser in the eyes with CRVO [130]. Currently, the laser photocoagulation in the case of RVO is considered the gold standard for complications such as retinal neovascularisation in the areas of retinal nonperfusion, reducing the risk of vitreous hemorrhage, as well as in the cases of iris neovascularisation [130,131].

### 5.3. Anti-VEGF Therapy

The use of anti-VEGF agents in macular edema secondary to RVO is supported by the robust evidence indicating that the intraocular levels of the VEGF are elevated in the patients with RVO as compared to the control group [132,133]. The activation of the VEGF pathway, secondary to hypoxia, induces neovascularisation and increases vascular permeability in the retina by directly affecting tight junctions and adherens junctions of the endothelium [30]. The anti-VEGF therapy is an effective therapeutic approach targeting the fundamental pathogenesis of macular edema in RVO, and currently, intravitreal injections of anti-VEGF agents have become the gold standard treatment [119,134]. The European Medicines Agency (EMA) and the United States Food and Drug Administration (FDA) have approved two anti-VEGF agents to be used for treating the macular edema secondary to the RVO: Ranibizumab (brand name Lucentis; manufactured by Genentech Inc., South San Francisco, CA, USA) and Aflibercept (brand name Eylea^®^; developed by Regeneron Pharmaceuticals Inc., Tarrytown, NY, USA, and Bayer HealthCare Pharmaceuticals, Berlin, Germany). At the end of October 2023, the FDA also approved Faricimab-svoa (marketed under the brand name Vabysmo™, developed by Genentech, San Francisco, CA, USA). Since 2022, Faricimab has been used for treating nAMD (neovascular Age-Related Macular Degeneration) and DME, which has been approved by both the FDA and EMA. Bevacizumab (marketed under the brand name Avastin; Genentech, South San Francisco, CA, USA/Roche, Basel, Switzerland) is additionally used in this indication beyond its registered indications.

#### 5.3.1. Ranibizumab

Ranibizumab is a recombinant humanised fragment of a monoclonal antibody IgG1 that binds to the vascular endothelial growth factor A and inhibits all of its isoforms. It is believed that the smaller size of that antibody facilitates penetration into the retina, although it may be associated with faster clearance from the vitreous body [135].

The BRAVO trial (Ranibizumab for the Treatment of Macular Edema following Branch Retinal Vein Occlusion: Evaluation of Efficacy and Safety) and CRUISE trial (Ranibizumab for the Treatment of Macular Edema following Central Retinal Vein Occlusion: Evaluation of Efficacy and Safety) are two multicentre, randomised, sham-controlled, double-masked clinical trials that have evaluated the efficacy and safety of intravitreal injections of Ranibizumab at the doses of 0.3 mg and 0.5 mg in the patients with macular edema secondary to the retinal vein occlusion [136,137]. In the BRAVO study, the focus was on the macular edema secondary to BRVO, while in the CRUISE study, the focus was on the macular edema secondary to CRVO. In both studies, a rapid and effective treatment of the macular edema was observed, with a low incidence of adverse events. In the BRAVO study, as a result of the 0.5 mg dose, the average treatment efficacy was +18.3 early treatment diabetic retinopathy study letters (ETDRS), with 61.1% of the patients experiencing an improvement of ≥15 letters or more, and the mean reduction in the central retinal thickness was 97.6%. Similarly, in the CRUISE study, with the same 0.5 mg dose, the average treatment efficacy was +14.9 ETDRS letters, with 47.7% of the patients experiencing an improvement of ≥15 letters or more, and the mean reduction in the central retinal thickness was 97.3%.

#### 5.3.2. Aflibercept

Aflibercept is a soluble receptor that binds to the VEGF-A, VEGF-B, and placental growth factor (PlGF) with a higher affinity than their natural receptors.

VIBRANT was a prospective, multicentre, double-masked, randomised, controlled phase 3 trial that evaluated the efficacy and safety of monthly intravitreal aflibercept (IAI) injections at the dose of 2 mg as compared to the grid laser in the patients with the macular edema secondary to the BRVO. The results were assessed at 24 and 52 weeks of the study. At week 24, there was an improvement of +17 ETDRS letters in visual acuity, and the percentage of the patients who had achieved an increase of > or =15 letters was 52.7% [138]. At 52 weeks of the study, the respective improvements were +17.1 ETDRS letters and 57.1%. The use of the laser therapy resulted in much less improvement. Furthermore, in the IAI group, only 10.6% of the patients required the rescue laser therapy at week 36, whereas in the laser/IAI group, as many as 80.7% required the rescue IAI from week 24 to week 48 [139].

COPERNICUS and GALILEO are two prospective, multicentre, double-blind, randomised, sham-controlled phase 3 trials that evaluated the efficacy and safety of monthly IAI at the dose of 2 mg as compared to the sham treatment in the patients with CRVO [140,141]. For 6 months, Aflibercept was administered every 4 weeks, and then from week 24 to week 48, the treatment group received it pro re nata. At the end of those two studies, 55–60% of the patients achieved at least a 15-letter improvement in vision on average, and the mean increase in ETDRS was +16.2 in the COPERNICUS study and +16.9 letters in the GALILEO study as compared to an improvement of +3.8 ETDRS in the untreated patients.

#### 5.3.3. Bewacizumab

Bevacizumab is a recombinant humanised monoclonal IgG1 antibody that prevents the binding of all isoforms of VEGF-A to endothelial cell receptors.

In the ophthalmic field, Bevacizumab is considered an effective drug for treating ME secondary to RVO. Since this drug has not been approved by the FDA for ophthalmic indications and is used off-label, clinical studies are not uniform [142,143].

The intravitreal administration of Bevacizumab every 6 weeks as compared to the sham treatment resulted in an improvement in visual acuity in 60% of the patients by a minimum of 15 letters, compared to 20% of the patients in the control group [144]. Two major reports from the American Academy of Ophthalmology confirmed the appropriateness of using Bevacizumab in the cases of ME secondary to BRVO and CRVO [145,146]. In the randomised clinical Study of Comparative Treatments for Retinal Vein Occlusion 2 (SCORE 2), the efficacy of intravitreally administered Bevacizumab (1.25 mg) was compared to Aflibercept (2 mg). Intravitreal Bevacizumab was not inferior to Aflibercept in terms of visual acuity after 6 months of treatment [147]. The follow-up of the patients at 12 months after the completion of the SCORE2 study did not show any differences in the visual acuity letter score (VALS) or the central subfield thickness (CST), regardless of the patient’s treatment assignment [148].

#### 5.3.4. Comparison of Ranibizumab, Aflibercept, and Bewacizumab

The treatment regimen for ME secondary to RVO for the three aforementioned preparations and the administered volume are the same. The recommended dose for Ranibizumab is 0.5 mg (0.05 mL), for Aflibercept it is 2 mg (0.05 mL), and for Bevacizumab it is 1.25 mg (0.05 mL) [149,150,151]. Treatment begins with one injection per month. After several loading doses (usually 3 or 4), some patients still require monthly injections, while others require the drug to be administered on an as-needed basis or with an extended dosing interval [149,150,151]. Patients showing no signs of disease activity require only follow-up examinations.

Although Ranibizumab and Bevacizumab are highly effective in neutralising VEGF, Ranibizumab exhibits 17 times greater binding affinity and 6 times greater binding affinity after extensive dilution [152]. Both of these drugs have a lower binding capacity as compared to Aflibercept. The dissociation constant of Aflibercept is 0.66, while for Ranibizumab and Bevacizumab, it is 20.6 and 35.1, respectively. This indicates that the binding affinity of Aflibercept is approximately 100 times greater than that of Ranibizumab and Bevacizumab [135]. Aflibercept effectively inhibited VEGF for a period of 7 days, Ranibizumab showed significant inhibition only for 72 h, while Bevacizumab lasted for only 48 h. Aflibercept has been recognised as the most effective in terms of long-lasting VEGF inhibition [153]. In the case of all the three drugs, the occurrence of serious systemic adverse events is rare, and the frequency of thromboembolic events and deaths for all the three drugs is similar [154].

#### 5.3.5. Faricimab

Faricimab is the first humanised, bispecific monoclonal antibody of immunoglobulin G, characterised by concurrent and independent binding to both the VEGF-A and angiopoietin-2 (Ang-2). It is believed to have a longer-lasting effect than the previous anti-VEGF drugs. This bispecific antibody also inhibits Ang-2, improving vessel stability and desensitising vessels to the effects of VEGF-A [155]. Moreover, numerous studies have shown that the dual inhibition of angiopoietin-2 and VEGF-A provides a more durable stabilisation of retinal vessels as compared to the exclusive inhibition of VEGF-A. An additional advantage of the drug may be the fact that the levels of Ang-2 are among the highest in the patients with RVO [156,157].

This drug could revolutionise the current treatment for the macular edema. Studies on exudative age related macular degeneration (AMD) and DME have shown that after four monthly doses of the drug, in the patients with no disease activity, consideration should be given to administering Faricimab every 16 weeks (4 months) [158,159]. In the case of DME, the interval may be extended by 4 weeks at a time. Importantly, there is no requirement for monthly visits to monitor the patient’s condition between injections.

The effectiveness of that antibody in the case of the ME secondary to the RVO has been demonstrated in the BALATON trial (ClinicalTrials.gov, identifier: NCT04740905) and COMINO trial (ClinicalTrials.gov, identifier: NCT0v4740931). In those two randomly assigned, multicentre, double-blind, phase 3 trials evaluating the efficacy and safety of Faricimab-svoa compared to Aflibercept, 553 patients with BRVO (BALATON) and 729 patients with CRVO or HRVO (COMINO) participated. Both BALATON and COMINO achieved their primary efficacy endpoint, with Faricimab demonstrating the non-inferior visual improvement as compared to Aflibercept at the main endpoint visit at 24 weeks [160].

In the BALATON study, the adjusted mean change in the BCVA as compared to the baseline at week 24 was +16.9 ETDRS letters in the Faricimab group compared to +17.5 ETDRS letters in the Aflibercept group [160]. In the COMINO study, it was +16.9 ETDRS letters for Faricimab and +17.3 ETDRS letters in the control group, respectively [160]. The reduction in CST accounted for by Faricimab was similar to that observed in the case of Aflibercept at all time points [160]. In both studies, Faricimab was well tolerated, and its safety profile was similar to that of Aflibercept. The most common complication, occurring in 3% of cases, was subretinal hemorrhage [160]. Moreover, a larger percentage of patients did not exhibit macular leakage at the 24-week mark when treated with Faricimab as compared to Aflibercept [160]. This observation may support the assumption that the dual-action of Vabysmo could provide additional benefits as compared to monospecific anti-VEGF antibodies.

In February 2024, Roche presented evidence that the visual improvement and reduction in retinal fluid achieved within the first 24 weeks of the study persisted up to 72 weeks [161].

The manufacturer recommends administering Faricimab for ME associated with RVO at a dose of 6 mg (0.05 mL of 120 mg/mL solution) intravitreally every 4 weeks, for 6 months [162].

### 5.4. Steroids

Corticosteroids act in an anti-inflammatory manner by inhibiting phospholipase A2, which leads to the blocking of the arachidonic acid pathway [163]. Blocking that pathway results in the reduced synthesis of thromboxanes, leukotrienes, and prostaglandins, which are mediators of inflammation. They also lower the concentration of chemokines and cytokines involved in the development of the macular edema in RVO—mainly MCP-1, IL-1, and Il-17 [164]. As a result, the tight junctions between retinal capillary cells are stabilised, reducing fluid accumulation in the retina and vascular permeability, which justifies their use in the cases of macular edema secondary to RVO. Additionally, their pleiotropic action involves the inhibition of the VEGF and neuroprotection [165].

Corticosteroids are second-line drugs due to their known complications, including cataract development and increased intraocular pressure [166,167]. The justification for using this group of drugs may include a lack of response to the anti-VEGF therapy despite having been administered a saturation dose. They can be considered as first-line agents in the patients who have recently experienced a serious cardiovascular event or are unable to attend monthly visits for the anti-VEGF drug administration [134].

#### 5.4.1. Triamcinolone

The SCORE-BRVO study compared the effectiveness and safety of intravitreal triamcinolone acetonide at doses of 1 mg and 4 mg with grid photocoagulation in the patients with the macular edema secondary to BRVO [168]. There was no difference in visual acuity after 12 months between the two study groups and the control group. The frequency of adverse events (especially increased intraocular pressure and cataracts) in the treatment group was high enough that, given the same efficacy in terms of visual acuity, laser treatment is recommended instead of any dose of intravitreal triamcinolone.

The SCORE-CRVO study had a similar design to the above study, with the control group consisting of individuals who did not receive treatment but were only observed for the natural course of ME secondary to CRVO [166]. The steroid administered intravitreally had an advantage over observation in the treatment of vision loss, and the 1 mg dose showed a better safety profile than the 4 mg dose. It was suggested to administer triamcinolone at a dose of 1 mg in the patients with a similar profile as in the clinical trial.

#### 5.4.2. Dexamethasone

In the Geneva study, the effectiveness of an implant containing Dexamethasone (Ozurdex) was evaluated for the ME secondary to BRVO and CRVO [167]. Treatment with the dexamethasone (DEX) implant was characterised by a beneficial safety profile over 12 months. An improvement of the BCVA by ≥15 letters as compared to the baseline was achieved in 30% and 32% of the patients 60 days after the first and second DEX implantation, respectively. Ozurdex is a sustained release implant, and its pharmacokinetics allow for maintaining high concentrations of Dexamethasone in the retina and vitreous body for the first 2–3 months post-injection, with lower concentrations persisting up to 6 months [169]. Multiple injections of the Ozurdex^®^ product in the patients with RVO are feasible and safe [170]. Another piece of evidence justifying the use of Dexamethasone in the case of RVO is the increase in the oxygen saturation difference measurement in the patients with RVO, indicating an improvement in retinal oxygenation [171]. In 2009, Ozurdex^®^ was approved by the FDA and EMA and was authorised for the treatment of adult patients with macular edema following RVO. 

### 5.5. Other Methods 

Other described therapeutic methods in the case of RVO include the use of systemic recombinant tissue plasminogen activator (rtPA), radial optic neurotomy, chorioretinal anastomosis, and arteriovenous sheathotomy, which are very rarely performed due to the high risk of complications that could lead to vision loss. Those complications include vitreous hemorrhage, visual field defects, or retinal detachment [55].

### 5.6. Combined Therapies

#### 5.6.1. Laser Photocoagulation and Anti-Vascular Endothelial Growth Factor Agents

Several studies have evaluated the impact of combined conventional laser photocoagulation (CLP) with anti-VEGF treatment in the management of ME secondary to BRVO. However, those studies have not demonstrated any significant benefits in terms of functional or anatomical improvement, or a reduction in the number of anti-VEGF therapies required for ME secondary to BRVO [172,173,174]. The results of the study by Yamamoto et al. demonstrated that the selective retina therapy (SRT), combined with anti-VEGF therapy in the cases of ME secondary to BRVO, may reduce the number of anti-VEGF treatments required without worsening the functional and anatomical outcomes as compared to the anti-VEGF therapy alone [175]. SRT selectively destroys the retinal pigment epithelium (RPE) without causing any thermal damage to surrounding tissues, which allows for the regeneration of the RPE during the healing process. It is thought to lead to the normalisation and reactivation of RPE cell functions, such as the regulation of VEGF production and pump function [176]. Recently, it has been demonstrated that adding the sub-threshold micropulse laser photocoagulation (SMLP) to the anti-VEGF therapy may also require fewer intravitreal injections than in the case of the anti-VEGF monotherapy, with equally good functional and morphological outcomes. SMLP demonstrates a greater safety than CLP and is as effective as CLP in treating ME associated with BRVO [177,178,179]. In their prospective, non-randomised study, Feng et al. found that SMLP may even be a better treatment strategy for the patients with the treatment-resistant ME secondary to BRVO, especially in the cases where the response is poor after three or more initial anti-VEGF injections, particularly in the patients with central macular thickness (CMT) ≤ 400 µm [180]. The action of SMLP involves generating multiple short-duration pulses with intervals between them, allowing for the cooling of the retina, thus protecting against damage to the RPE or photoreceptors. The biological consequence is the stimulation of the RPE to produce “heat shock proteins” (HSP), the inhibition of cytokine production responsible for neovascularisation, mainly VEGF, as well as the restoration of Müller cell function and antioxidant-oxidant balance [181].

#### 5.6.2. Anti-VEGF Agents and Steroids

There are no controlled studies evaluating the effectiveness of such a combination therapy. There is some limited evidence that the combination therapy with anti-VEGF medication and DEX implant leads to an extended interval between subsequent injections as compared to the anti-VEGF monotherapy [182,183]. The patients with ME secondary to BRVO, especially, showed a more significant improvement in the BCVA and a reduction in the CRT at various time intervals [184]. When choosing this method, one should be prepared for a slight increase in the intraocular pressure (IOP), but usually, this side effect can be managed with topically applied medications [184].

### 5.7. Treatment of Neovascular Glaucoma in the Course of Ischemic CRVO

Hypoxia of the tissues and the pathological neovascularisation of the anterior segment of the eye lead to NVG, resulting in increased intraocular pressure and glaucomatous optic neuropathy [185]. The main promoter of the NVG development is VEGF, and its concentration is much higher in ischemic CRVO than in non-ischemic CRVO [186]. VEGF-targeted therapies for NVG include panretinal photocoagulation (PRP) and anti-VEGF injections. 

In the case of the NVG, strict patient monitoring is very important. The neovascularisation of the anterior segment in a patient diagnosed with ischemic CRVO typically occurs within the first 6–7 months, and over 80% of NVG cases develop within 6–8 months of the onset of the disease [187]. PRP is recommended only after the neovascularisation of the iris becomes apparent [134]. In exceptional situations, when systematic patient observation is not feasible, early prophylactic PRP is recommended in ischemic CRVO within the first 90 days of the onset of the disease [134].

The intravitreal anti-VEGF functionally delays the onset of neovascularisation as compared to the natural course of the disease in the patients with ischemic CRVO, but it does not prevent its occurrence [188]. For this reason, anti-VEGF agents are considered an adjunctive therapy. When a high IOP develops, it is difficult to achieve the desired therapeutic effect of PRP on NVG [189]. Treatment with anti-VEGF agents can effectively control the IOP within 1 month, but over a longer period of observation, it does not show such an effect [190]. There is evidence for the effective long-term treatment of NVG by means of a combination therapy consisting of PRP and angiogenesis inhibitors [191,192,193]. In some cases, intracameral injections of anti-VEGF are suggested to achieve faster and more accurate therapeutic outcomes [194].

If PRP and anti-VEGF therapies are ineffective, conventional glaucoma medications and surgical procedures are recommended. Surgical procedures include trabeculectomy, glaucoma drainage devices, and minimally invasive glaucoma drainage procedures. Refractory glaucoma can be treated with cyclophotocoagulation or cyclocryotherapy, which involve reducing the production of aqueous humour by deliberately damaging the ciliary body.

### 5.8. Follow-Up Recommendations

Due to the higher risk of complications in the case of CRVO and the possibility of converting NI-CRVO into I-CRVO, more frequent monitoring is recommended. In the case of treatment with anti-VEGF drugs, the frequency of follow-up appointments depends on the chosen drug and treatment scheme, such as “pro re nata” or “treat-and-extend”. For the patients with I-CRVO who have completed the anti-VEGF therapy for ME, as well as those complicated by the neovascularisation of the anterior segment, monthly monitoring is recommended in the first year to detect the onset of neovascularisation [120] (Table 5). For the patients with significant ischemia but without current complications, monthly check-ups are recommended for the first 6 months, followed by check-ups every 3 months for 1 year. In uncomplicated cases, check-ups are typically not required after 3 years from the onset of the condition [120].

In the case of BRVO, there are no strictly defined timeframes for patient follow-up appointments [119,120]. Follow-up visits at intervals of 3–4 months are recommended for the patients with ischemia involving one quadrant of the retina or more. In situations where the treatment targeting ME needs to be initiated, the choice of medication and treatment regimen depends on individual considerations.

### 5.9. Pharmacoeconomics

Restoring and maintaining vision in the case of RVO typically depends on frequent and costly therapies and numerous follow-up visits. Such a significant burden, coupled with the costs of various therapeutic options, raises socio-economic questions. The cost of treating BRVO with the macular edema in terms of dollars per quality-adjusted life year (QALY) ranges from approximately USD 800 to USD 26,000, while in the case of CRVO with macular edema it ranges from about USD 1400 to USD 16,000. Calculations for BRVO, depending on the therapeutic method used, showed that the Dollars per QALY ratio amounted to USD 824 for Bevacizumab, USD 1572 for the grid laser, USD 5536 for Ozurdex, and USD 25,566 for Ranibizumab [195]. Yet another analysis, comparing Bevacizumab with Aflibercept, has shown that Bevacizumab is both cheaper and more effective than Aflibercept in terms of the QALY [196]. Participants who had started treatment with Bevacizumab incurred the cost of USD 3213 in the first year of treatment, with their QALY increasing by 0.026. For Aflibercept, the corresponding cost amounted to USD 21,340 with the QALY increased by 0.020.

Economic considerations in healthcare always raise doubts about whether it is ethical to prioritise quality of life over its length. However, it has been shown that the RVO is an indicator of the worsening of the overall vascular status and an increased use of medical resources over a longer period of time [197].

## 6. Future Directions

As has been demonstrated above, the therapeutic possibilities for treating RVO mainly rely on RVO prevention and limiting the disease progression. RVO is a significant cause of vision impairment of retinal origin, which motivates researchers to continuously develop pharmacological interventions in this area.

Research is underway to introduce new drugs with a mechanism of action similar to anti-VEGF agents but with prolonged efficacy, such as bioconjugate drugs like KSI-301 (NCT04592419), as well as new methods of delivering existing substances. This group includes transscleral, controlled-release dexamethasone delivered via episcleral reservoir (NCT04120311), dexamethasone implant IBE-814 (NCT04576689), and Port Delivery System (PDS) [198]. The Port Delivery System (PDS) is a durable implant with the ability for multiple refills, which is inserted into the eye through a small incision in the sclera. The drug present in the PDS passively diffuses according to the concentration gradient from the implant reservoir to the vitreous cavity. The FDA has already approved the PDS implant containing Ranibizumab (SUSVIMO) for its use in the patients with nAMD, who have shown a positive response to the first two anti-VEGF injections. The Archway study demonstrated that 98.4% of patients treated with the PDS containing Ranibizumab did not require any supplemental treatment within the first 24 weeks [199]. The system is currently being investigated with respect to Faricimab as well (NCT04567303).

Currently, phase I/II clinical trials are underway regarding intravitreal autologous CD34+ stem cell therapy for the treatment of central retinal vein occlusion (NCT03981549). CD34+ stem cells in human bone marrow are mobilised into circulation in response to tissue ischemia for revascularisation and repair. Previous studies have shown the intraretinal migration of CD34+ cells and the presence of receptors for these cells on vascular endothelial cells, indicating their revascularisation potential [200]. In phase I, there is another study involving the same therapy, where the study group comprises the patients with loss of visual function due to various retinal degenerations and retinopathies, including CRVO/BRVO (NCT01736059).

Gene therapies offer an attractive, alternative approach, potentially opening the door to minimally invasive, one-time treatments. The technique involves using an adenovirus vector to deliver the anti-VEGF gene, thereby preventing the development of choroidal neovascularisation (CNV) in an inducible manner after a single intravitreal injection, and it shows promising results [201]. Gene therapy protocols involving the delivery of Aflibercept and Ranibizumab are currently in clinical trial phases. ADVM-022 is an AAV-7m8 vector encoding Aflibercept, which is in phase II clinical trials aimed at evaluating the safety, tolerability, and efficacy of a single injection in the patients with exudative AMD (NCT05536973). RGX-314 (NCT05407636), which expresses a fragment of a monoclonal antibody similar to Ranibizumab, is in phase III clinical trials and is being developed as a potential one-time treatment for exudative AMD. Both of those agents, by targeting the VEGF signalling pathway, may be used in the future for treating the RVO. Currently, a phase I study is evaluating the safety and efficacy of the VEGFA-targeted gene therapy in treating refractory neovascular retinal and choroidal diseases, including ME secondary to RVO (NCT05099094).

Alternative strategies for blocking angiogenesis independently of VEGF are needed. Huang presented a positive outcome of an anti-angiogenic gene therapy protocol that selectively targets pathological angiogenesis through a mechanism independent of VEGF. He confirmed the safety and efficacy of anti-Scg3 gene therapy in treating nAMD in a mouse model [202]. There are considerations underway for the selective activation of the Wnt signalling pathway or the utilisation of long non-coding RNAs (lncRNA) transcripts in retinal vascular diseases [203,204].

The new formulation developed by Avrutsky et al. may prove to be a more effective and convenient method for treating RVO compared to anti-VEGF drugs. They have patented a droplet formulation containing a caspase-9 inhibitor. In their study, based on a mouse model of RVO, they demonstrated that the rate of reperfusion of occluded retinal veins using the caspase-9 inhibitor, as well as the reduction of hyperreflective foci, was comparable to anti-VEGF drugs. Moreover, inhibiting caspase-9 increased the reduction of retinal edema twice as much as anti-VEGF drugs, and only the neutralisation of caspase-9 reduced retinal atrophy, indicating neuroprotection [205]. Caspase-9 is an initiator of the intrinsic apoptotic pathway and is involved in various inflammatory and degenerative pathologies, both through apoptotic and non-apoptotic mechanisms [206]. Recently, it has been identified as a new mediator of retinal damage in RVO by initiating neurovascular dysfunction and regulating edema and gliosis. The results of Avrutsky’s study demonstrate the justification for the development of RVO therapies that are not solely focused on VEGF. The topical form of drug delivery appears to be much more patient-friendly as compared to intravitreal injections.

The development of medicine is closely linked to the advancement of new technologies, including artificial intelligence (AI) and machine learning (ML). Thanks to those tools, we are able to assess various data, including imagery that may not be accessible to the naked eye of an ophthalmologist. It would not be possible without easy access to large datasets of ophthalmic data, which, as Khan et al. calculated, amount to 94 datasets comprising 507,724 images [207]. 

Examining the fundus of the eye provides one of the easiest insights into the human vascular system, and the structure of the retinal vessels can be considered a reflection of the systemic vascular condition. The development of this field of medicine has led to the emergence of a new approach called ‘oculomics’ that aims to search for retinal vascular biomarkers, increasingly utilising artificial intelligence algorithms in combination with retinal image datasets [208]. It is not surprising, therefore, that there is evidence indicating that a fundus photograph can be a source of information such as age, gender, blood pressure, smoking status, or the body mass index of the patient. Those data are among the risk factors for cardiovascular disease. This has led to a point where an AI/ML algorithm trained on fundus photographs can identify patients at high risk of myocardial infarction [209,210].

In the field of ophthalmology, AI technology has made incredible advances in the diagnosis of retinal diseases such as diabetic retinopathy, retinopathy of prematurity, or age-related macular degeneration [211,212,213,214]. Indeed, it is interesting to note that autonomous AI/ML medical devices for detecting diabetic retinopathy are already available in the American and European markets [215,216]. Recently, large language models (LLMs), mainly the Chatbot Generative Pre-trained Transformer (ChatGPT), have greatly developed, allowing for faster acquisition of medical knowledge and the determination of patient groups with eye diseases after conducting a subjective examination. It provides hope for improving diagnostic and research capabilities for eye diseases [217].

In the case of RVO, diagnostic models utilising AI/ML algorithms trained on fundus images are available. Those models diagnose various types of RVO as effectively as retina specialists, potentially relieving clinicians of the burden of screening for RVO, thus increasing their efficiency in managing patients already diagnosed with the condition [218,219]. Miao et al. developed a different deep learning (DL) model that was able to detect the type of ischemia and areas of non-perfusion based on colour fundus photographs of the retina, thereby predicting the need for laser photocoagulation in the cases of BRVO almost as effectively as experienced specialists, and significantly better than residents [220]. The study by Gallardo et al. even showed that it was possible to predict through machine learning the individual need for the anti-VEGF therapy, especially in the patients with “low-demand” vascular occlusion, with IRF being the most important biomarker [221]. Schlosser et al. presented an interesting study, in which they demonstrated that by leveraging machine learning (ML) and deep learning (DL), it was possible to make predictions regarding BCVA in the patients with retinal diseases, including RVO, with an accuracy level of 69%, placing them in the same range as retina specialists [222].

## 7. Conclusions

Retinal vein occlusion is a significant cause of vision loss and a crucial predictor of cardiovascular disease, which has led to a large body of research analysing this condition. However, the diagnosis and treatment of RVO have changed dramatically in recent years, requiring clinicians to continuously update their knowledge. New imaging techniques allow for a better understanding of the pathophysiology of the disease, retinal anatomy, and the mechanisms responsible for the macular edema formation. Thanks to this development, medicine can provide much faster diagnostics for RVO, directly impacting treatment outcomes. The RVO therapy remains a significant challenge due to the multifactorial nature of this disease entity. A wide range of therapeutic options support an individualised approach to achieve satisfactory results and avoid complications. Exploring new therapeutic options, such as Faricimab, may reduce the frequency of burdensome treatments, leading to a positive psychological impact. Improving currently available therapies, developing new therapeutic options, and increasing the access to AI will reduce the burden associated with this disease. The WHO World Report on Vision observes that ‘Recent scientific and technological developments promise to further accelerate advances. Nonetheless, progress is not keeping pace with population eye care needs’ [223,224]. Leveraging new technologies intelligently in order to address the global issue of visual impairment, including RVO, should be a health priority. In the case of RVO, preventive measures should not be overlooked, and collaboration between ophthalmologists and primary care physicians is essential for coordinating the treatment of systemic diseases.

## 8. Method of Literature Search

Peer-reviewed journals were the source of all articles we had considered, and pertinent articles have been found in ScienceDirect, Web of Science, and PubMed databases. These databases were searched with no year limitations. The following keywords were used: “treatment”, “diagnosis” and “macular edema”. Each of these keywords was used in combination with the expression “retinal vein occlusion”. Further searches were conducted combining the stated keywords with epidemiology, mechanisms, pathogenesis, neovascularisation, and AI. After reviewing the available literature, we included relevant information, updating and consolidating the knowledge about retinal vein occlusion. No automation tools or machine learning techniques were used in this research.

## Figures and Tables

**Table 2:** Major biological factors detected in the vitreous humor in patients with cystoid macular edema due to retinal vein occlusion [30].

CRVO	IL-1β, IL-6, IL-8, TGF-β, VEGF, bFGF, CXCL-10 or IP10, CCL2 or MCP-1, PDGF-AA, SAA
BRVO	IL-1β, IL-6, IL-8, IL-12, IL-15, IL-17, IL-2, TGF beta, VEGF, sVEGFR2, bFGF, sICAM-1 or CD54, CCL2 or MCP-1, sVEGFR, SAA

**Table 3:** Clinical features characteristic for acute and late stages of RVO [55].

Acute RVO	Chronic RVO
dilated, tortuous retinal veins intraretinal hemorrhages cotton wool spots optic disc edema retinal edema	intraretinal hard exudates shunt vessels cystoid macular edema epiretinal membrane neovascularisation of retina and/or optic disc and/or rubeosis iridis complications of neovascularisations: vitreous hemorrhages, retinal detachment, neovascular glaucoma

**Table 4:** Tests for RVOs [119,120].

Routine Investigations	Additional Investigations
▪ systolic blood pressure ▪ diastolic blood pressure ▪ fasting serum levels of glucose and glycated hemoglobin ▪ fasting levels of lipids ▪ complete blood count ▪ renal function test	▪ homocysteine levels ▪ protein C and protein S levels ▪ antithrombin III levels ▪ factor XII levels ▪ PCR for the detection of human Factor V Leiden G1691A mutation ▪ PCR for the detection of the human Factor II (Prothrombin) G20210A mutation ▪ antiphospholipid antibodies—lupus anticoagulant and anticardiolipin antibodies ▪ treponemal and nontreponemal tests ▪ Mantoux test ▪ QuantiFERON test

**Table 5:** Follow-up recommendations for I-CRVO [120].

Eye Condition in I-CRVO	Time of Monitoring
completed anti-VEGF therapy due to macular edema	monthly in the first year
neovascularisation of the anterior segment	monthly in the first year
significant ischemia without complications	monthly for the first 6 months, followed by check-ups every 3 months for 1 year

## Abbreviations

AI	artificial intelligence
AKT	protein kinase B
AMD/nAMD	Age-Related Macular Degeneration/neovascular Age-Related Macular Degeneration
AMPK	AMP-activated protein kinase
Ang-2	angiopoietin-2
AQP4	aquaporin 4 channels
BA	bile acids
BCVA	best-corrected visual acuity
BRVO	branch retinal vein occlusion
BVOS	Branch Vein Occlusion Study
bFGF	basic fibroblast growth factor
CCL2	chemokine ligand 2
CD54	cluster of differentiation 54
CLP	conventional laser photocoagulation
CME	cystoid macular edema
CMT	central macular thickness
CRP	c-reactive protein
CRT	central retinal thickness
CRVO	central retinal vein occlusion
CVOS	Central Vein Occlusion Study
CXCL-10	C-X-C motif chemokine 10
DDD	defined daily doses
DEX	dexamethasone
DME	diabetic macular edema
DRIL	disorganisation of retinal inner layers
EC	endothelial cell
EMA	European Medicines Agency
ETDRS	Early Treatment Diabetic Retinopathy Study
EZ	ellipsoid zone
FA	fluorescein angiography
FAK	focal adhesion kinase
FAZ	foveal avascular zone
FDA	The United States Food and Drug Administration
FMT	fecal microbiota transplantation
GABA	gamma-aminobutyric acid
HF	hyperreflective foci
HIF-1	hypoxia-inducible factor-1
HRVO	hemi-retinal vein occlusion
IAI	intravitreal aflibercept
I-CRVO	ischemic central retinal vein occlusion
IDA	iron deficiency anemia
IGF-1	insulin-like growth factor 1
IL	interleukin
IOP	intraocular pressure
IP10	interferon gamma-induced protein 10
IRF	intraretinal fluid
ISI	ischemic index
LMWH	low molecular weight heparins
lncRNA	long non-coding RNAs
LPA-ATX	lysophosphatidic acid-autotaxin
LPS	lipopolysaccharides
MAPK	mitogen-activated protein kinase
MCP-1	monocyte chemoattractant protein 1
ME	macular edema
MMP	metalloproteinase
NLR	neutrophil-to-lymphocyte ratio
NI-CRVO	non-ischemic central retinal vein occlusion
NOAC	non-vitamin K-antagonist oral anticoagulant
NV	neovascularisation
NVG	neovascular glaucoma
OCT	optical coherence tomography
OCTA	optical coherence tomography angiography
OLM	outer limiting membrane
PAI-1	plasminogen activator inhibitor-1
PAMM	severe acute macular neuroretinopathy
PC	pericytes
PDGF-AA	platelet derived growth factor
PDS	Port Delivery System
PEDF	pigment epithelium-derived factor
PlGF	placental growth factor
PI3K	phosphatidylinositol 3-*kinase*
PLC-γ	phospholipase C gamma
PLR	platelet-to-lymphocyte ratio
PRP	panretinal photocoagulation
PVD	posterior vitreous detachment
QALY	quality-adjusted life year
RMG	Müller glial cells
RPE	retinal pigment epithelium
rtPA	systemic recombinant tissue plasminogen activator
RVO	retinal vein occlusion
SAA	serum amyloid A
SCFA	short-chain fatty acids
sICAM-1	soluble intercellular adhesion molecule 1
SMLP	sub-threshold micropulse laser photocoagulation
SRD	serous retinal detachment
SRF	subretinal fluid
SRT	selective retina therapy
SVD	small vessel disease
sVEGFR2	soluble vascular endothelial growth factor receptor 2
TGF-β	transforming growth factor β
TMAO	trimethylamine N-oxide
TNF	tumor necrosis factor
TRD	tractional retinal detachment
TUDCA	tauroursodeoxycholate
UWF-FA	ultra-widefield fluorescein angiography
VA	visual acuity
VALS	visual acuity letter score
VEGF	vascular endothelial growth factor
VH	vitreous hemorrhage
ZO-1	zonula occludens-1
